# Rapidly decreased HBV RNA predicts responses of pegylated interferons in HBeAg-positive patients: a longitudinal cohort study

**DOI:** 10.1007/s12072-020-10015-3

**Published:** 2020-02-25

**Authors:** Min Zhang, Guangdi Li, Jia Shang, Chen Pan, Minxiang Zhang, Zhibiao Yin, Qing Xie, Yanzhong Peng, Qing Mao, Xinqiang Xiao, Yongfang Jiang, Kaizhong Luo, Yun Xu, Hai Ding, Wenzhou Fan, Vidaurre Diego, Mahmoud Reza Pourkarim, Erik De Clercq, Guiqiang Wang, Guozhong Gong

**Affiliations:** 1 Institute of Hepatology and Department of Infectious Diseases, The Second Xiangya Hospital, Central South University, Changsha, 410011 Hunan China; 2 Department of Epidemiology and Health Statistics, Xiangya School of Public Health, Central South University, Changsha, 410078 Hunan China; 3 Department of Infectious Diseases, Henan Provincial People’s Hospital, Zhengzhou, 450003 Henan China; 4 Department of Gastroenterology, Mengchao Hepatobiliary Hospital of Fujian Medical University, Fuzhou, 350025 Fujian China; 5 Department of Infectious Diseases, The Sixth People’s Hospital of Shengyang, Shengyang, 110006 Liaoning China; 6 Department of Infectious Diseases, Guangzhou Eighth People’s Hospital of Guangzhou Medical University, Guangzhou, 510260 Guangdong China; 7 Department of Infectious Diseases, Rui-Jin Hospital, Shanghai Jiao Tong University School of Medicine, Shanghai, 200025 China; 8 Department of Infectious Diseases, Peking University Shenzhen Hospital, Shenzhen, 518036 China; 9 Chongqing Key Laboratory for Research of Infectious Diseases, Department of Infectious Diseases, Southwest Hospital, Third Military Medical University, Chongqing, 400038 China; 10 Hunan Sansure Biotech Incorporation, Changsha, 410205 Hunan China; 11 Oxford Centre for Human Brain Activity, University of Oxford, Oxford, OX3 7JX UK; 12 Department of Microbiology, Immunology and Transplantation, Division of Clinical and Epidemiological Virology, KU Leuven, 3000 Leuven, Belgium; 13 Health Policy Research Center, Institute of Health, Shiraz University of Medical Sciences, Shiraz, Iran; 14 Department of Microbiology and Immunology, Rega Institute for Medical Research, KU Leuven, 3000 Leuven, Belgium; 15 Department of Infectious Diseases, The Center for Liver Diseases, Peking University First Hospital, Beijing, 100034 China

**Keywords:** Hepatitis B, HBV RNA, Pegylated interferon alfa, HBeAg seroconversion, Antiviral treatment

## Abstract

**Background:**

As an important anti-HBV drug, pegylated interferon α (PegIFNα) offers promising clinical efficacy, but biomarkers that accurately forecast treatment responses are yet to be elucidated. Here, we evaluated whether HBV RNA could act as an early monitor of pegylated interferon responses.

**Methods:**

We analyzed a phase 3, multicenter, randomized cohort of 727 HBeAg-positive non-cirrhotic patients receiving a 48-week treatment of PegIFNα-2a or PegIFNα-2b and a 24-week treatment-free follow-up. Serum levels of HBV RNA, HBV DNA, HBeAg, and HBsAg were measured at weeks 0, 12, 24, 48, and 72.

**Results:**

HBeAg seroconversion and HBsAg loss at week 72 were observed in 217 (29.8%) and 21 (2.9%) patients, respectively. During the 48-week treatment, HBV RNA decreased more rapidly than HBV DNA and HBsAg, but HBV RNA and HBeAg shared similar dynamics with positive correlations. Multivariate regression analyses consistently revealed the significance of HBV RNA at weeks 0, 12, 24, and 48 to monitor HBeAg seroconversion but not HBsAg loss. Although baseline HBV RNA only showed a modest AUC performance, HBV RNA with a significant increase of AUC at week 12 outperformed other HBV biomarkers to forecast HBeAg seroconversion (*p* value < 0.05). HBV RNA ≤ 1000 copies/mL was an optimized cutoff at week 12 that offered better prediction than other HBV biomarkers. This optimized cutoff plus patient age, HBV genotype B, and HBeAg offered a strong estimation of HBeAg seroconversion (accuracy 95.2%, true negative rate 99.8%).

**Conclusion:**

HBV RNA at week 12 is an effective monitor of HBeAg seroconversion in HBeAg-positive patients treated with pegylated interferons.

**Electronic supplementary material:**

The online version of this article (10.1007/s12072-020-10015-3) contains supplementary material, which is available to authorized users.

## Introduction

To offer effective treatment responses towards hepatitis B virus (HBV) infections, eight FDA-approved drugs belonging to two classes are currently available, including two interferon alfa drugs and six nucleos(t)ide analogs [1]. HBeAg seroconversion rates were generally higher in HBeAg-positive patients receiving interferon therapies than nucleos(t)ide analogs. To offer better treatment responses, the assessment of pre-treatment viral factors (e.g., HBV genotypes), on-treatment viral factors (e.g., HBV DNA, HBsAg), host and environmental factors (e.g., patient age, ALT) is recommended for the initiation and continuation of HBV therapies [2–4]. Nevertheless, no single biomarker has been recognized to fully predict treatment responses [5, 6].

HBV RNA has been recently proposed as a new biomarker to assess treatment responses, because HBV RNA transcripts produced directly from HBV cccDNA may monitor the progressive clearance of HBV cccDNA reservoirs [7–9]. As a potential predictor of treatment responses, serum HBV RNA is associated with intrahepatic cccDNA levels [10] and the persistence of viral infection and rebound [11]. For instance, an early decrease of HBV RNA effectively predicted HBeAg seroconversion in 62 patients receiving either lamivudine or tenofovir [12]. In a responder-enriched subpopulation of 76 patients treated with PegIFNα-2a, HBV RNA was recognized as a sound predictor of HBeAg seroconversion by univariate analyses alone [13], whereas its strength was unclear in large cohorts with non-responders.

Despite encouraging findings above, many aspects of HBV RNA remain unclear. First, dynamic changes of HBV RNA in large-scale cohorts of interferon-treated patients remain poorly understood, since previous studies focused mostly on nucleos(t)ide analogs. Second, it is ambiguous if HBV RNA acted as an effective predictor of interferon responses, particularly in a large cohort with non-responders. Third, to offer better responses, it remains unclear whether HBV RNA in combination with known HBV biomarkers could offer better predictive performance. Last, it remains unclear whether HBV RNA could predict HBsAg loss which is generally considered as a functional “cure” that signifies a favorable outcome of antiviral treatments [14, 15]. To address the above questions, we assessed HBV RNA in a longitudinal cohort of 727 HBeAg-positive patients who randomly received PegIFNα-2a or PegIFNα-2b. Based on this large-scale cohort, our findings support the hypothesis that even at the early interferon treatments, HBV RNA is a strong monitor of HBeAg seroconversion, despite its modest role in the prediction of HBsAg loss.

## Materials and methods

### Patients

**Fig. 1:**
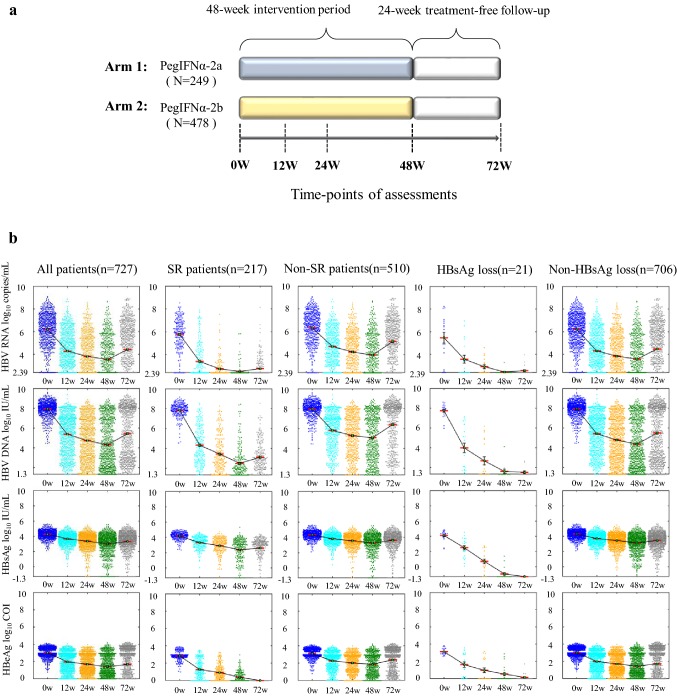
Study profile (**a**) and distributions of HBV biomarkers throughout 72 weeks (**b**). Scatter plots of HBV RNA, HBV DNA, HBsAg, and HBeAg were shown in five groups: (1) all patients; (2) SR patients who achieved HBeAg seroconversion; (3) Non-SR patients who failed to achieve HBeAg seroconversion; (4) HBsAg loss patients who achieved HBsAg loss; and (5) non-HBsAg loss patients who failed to achieve HBsAg loss. Mean values were linked by red lines

Between March 2013 and July 2015, a total of 855 HBeAg-positive non-cirrhotic patients who were infected with HBV mono-infection and treatment-naïve for at least 6 months were treated with either PegIFNα-2a or PegIFNα-2b in a phase 3, multicenter, randomized, controlled clinical trial [16]. Patients were randomly assigned in a ratio of 1:2 to receive PegIFNα-2a (Pegasys^®^, Roche, Switzerland) or PegIFNα-2b (PegBeron^®^, Amoytop Biotechnology, China) at the dose of 180 μg/week for 48 weeks and a treatment-free follow-up for 24 weeks (Fig. 1a, Figure S1). PegBeron^®^ was approved by the China Food and Drug Administration in 2017. This study was performed using the cohort of 727 patients who completed the full course of the randomized trial and had serum samples at week 72 to measure treatment responses. This trial was registered at ChinaDrugTrials.org (ID: TB1211IFN) and ClinicalTrials.gov (NCT01760122). Inclusion and exclusion criteria were described in our clinical protocol (see Supplementary).

### Laboratory assessments

Routine physical examinations with biochemical and hematologic assessments were conducted at weeks 0, 12, 24, 48, and 72. HBV RNA was extracted from patient serum samples (200 μL) using a nucleic acid extraction kit (Sansure Biotech Inc. China) which was developed based on the magnetic bead technology [17]. Eluted HBV RNA (10 μL) obtained from HBV RNA extraction was used for reverse transcription. Primers of HBV RNA-RACE, HBV RNA-forward, HBV RNA-reverse, and HBV RNA-probe (see Supplementary) that target conserved regions of the HBV genome were obtained from the literature [12]. Armored RNA internal controls were added during sample lysis [18]. In the absence of DNA polymerase and cDNA primers, HBV RNA was reverse transcribed into cDNA under the temperature of 50 °C for 30 min. After adding DNA polymerases and cDNA primers, the cDNA amplification was performed by an activation step at 95 °C for 2 min, followed by 50 two-step cycles (each cycle 15 s at 95 °C and 30 s at 60 °C), and a cooling step down to 25 °C for 10 s. The fluorescence of cDNA was detected and measured by the 7500 Real-Time PCR System (Applied Biosystems^®^). More details are provided in our Supplementary.

Elecsys tests (Roche Diagnostics GmbH, Germany) were used to measure HBeAg, anti-HBe, HBsAg, and anti-HBs. HBV DNA was quantified by the Roche Diagnostics Cobas^®^ Amplicor HBV Test, Version 2.0 (Roche Diagnostics, Germany). ALT assay was conducted using Architect c8000 clinical chemistry analyzer (Abbott, USA) with the IFCC standard for enzyme determination. The NCBI HBV genotyping tool [19] was applied to determine HBV genotypes with inputs of HBV polymerase sequences extracted by conventional Sanger sequencing. Detection limits of HBV RNA, HBV DNA, HBeAg, and HBsAg were 250 copies/mL, 20 IU/mL, 1 COI (cut of index), and 0.05 IU/mL, respectively. The log_10_ values of four biomarkers above were transformed prior to statistical analyses.

### Definitions of treatment responses

HBeAg seroconversion, also called serological response (SR), was defined as the disappearance of HBeAg accompanied by the gain of anti-HBe throughout the treatment-free follow-up [20]. HBsAg loss (HL) indicated the disappearance of HBsAg [14].

### Statistical analyses

Four major statistical analyses were performed in our study. First, to explore statistical differences of biomarker values between patient groups, Fisher’s exact tests and Mann–Whitney tests were conducted for categorical and continuous variables, respectively. Logistic regressions were performed to estimate the odds ratio (OR) and 95% confidence intervals (95% CI). Second, we quantified correlations between continuous biomarkers using Spearman correlation coefficients. Third, logistic linear regression analyses revealed factors significantly associated with treatment responses. Predictive performance was measured by AUC analyses, while HBV biomarker values below detection limits were replaced with their detection limits. Fourth, a standard software, Cutoff Finder, was applied to optimize biomarker cutoffs [21]. The standard bootstrap resampling method was applied to generate 1000 randomized datasets in the fivefold cross-validation to avoid overestimation. The robustness of biomarker cutoffs was confirmed by bootstrapping resampling and cross-validation. Sixth, predictor importance estimates were measured by random forest classification (500 decision trees) with curvature tests for splitting predictors and surrogate splits for handling missing values. Since missing data only appeared in 1.1% of our dataset, our analyses used all available cases when variables of interest were present—a known approach called pairwise deletion. Analyses were conducted using MATLAB R2016a.

## Results

### Demographic profiles and baseline characteristics

Our entire cohort consisted of 523 males and 204 females predominately infected with HBV genotype C (*n* = 427, 58.7%), followed by genotypes B (*n* = 292, 40.2%), D (*n* = 7, 1%), and B/C recombinant (*n* = 1, 0.1%). At baseline, the mean and standard error of patient age, body weight, and ALT were 28.2 ± 0.3 years, 63.6 ± 0.4 kg, and 193.0 ± 5.5 IU/mL, respectively (Table 1). Moreover, baseline values of HBV RNA, HBV DNA, HBsAg, and HBeAg were 6.2 ± 0.05 log_10_ copies/mL, 7.9 ± 0.03 log_10_ IU/mL, 4.3 ± 0.02 log_10_ IU/mL, and 3.0 ± 0.02 log_10_ COI, respectively. Distributions of HBV biomarkers are shown in Fig. 1b.

**Table 1:** Baseline characteristics of host and HBV biomarkers in our study

	All patients (*n* = 727)	SR (*n* = 217)	Non-SR (*n* = 510)	*p* value	HBsAg loss (*n* = 21)	HBsAg positive (*n* = 706)	*p* value
Age (years)	28.2 ± 0.3	26.7 ± 0.4	28.8 ± 0.3	5.8 × 10^−4^	27.6 ± 1.3	28.2 ± 0.3	0.98
Male gender	523 (71.9%)	150 (69.1%)	373 (73.1%)	0.27	11 (52.4%)	512 (72.5%)	0.04
Body weight	63.6 ± 0.4	61.7 ± 0.7	64.4 ± 0.5	0.03	57.9 ± 2.6	63.7 ± 0.4	0.11
ALT (IU/mL)	193.0 ± 5.5	211.8 ± 10.1	184.9 ± 6.5	0.003	196.4 ± 25.8	192.9 ± 5.6	0.9
HBV genotypes				3 × 10^−4^			0.4
B	292	113	179		12	280	
C	427	103	324		9	418	
D	7	1	6		0	7	
B + C	1	0	1		0	1	
HBV RNA^#^	6.2 ± 0.05	5.8 ± 0.10	6.4 ± 0.06	2 × 10^−5^	5.5 ± 0.5	6.2 ± 0.05	0.11
HBV DNA^#^	7.9 ± 0.03	7.8 ± 0.05	8.0 ± 0.03	2 × 10^−4^	7.7 ± 0.2	7.9 ± 0.03	0.16
HBsAg_#_	4.3 ± 0.02	4.2 ± 0.03	4.3 ± 0.02	6 × 10^−5^	4.1 ± 0.1	4.3 ± 0.02	0.19
HBeAg^#^	3.0 ± 0.02	2.9 ± 0.04	3.1 ± 0.02	3 × 10^−9^	3.1 ± 0.09	3.0 ± 0.02	0.49
PegIFNα-2a/PegIFNα-2b	249/478	66/151	183/327	0.16	5/16	244/462	0.32

^#^The log_10_ transformation was performed prior to analyses. Biomarker units are measured by log_10_ copies/mL for HBV RNA, log_10_ IU/mL for HBV DNA, log_10_ IU/mL for HBsAg, and log_10_ COI for HBeAg

HBeAg seroconversion and HBsAg loss at week 72 were observed in 217 (29.8%) and 21 (2.9%) patients, respectively. HBeAg seroconversion was found in 26.5% (66/249) of PegIFNα-2a-treated patients, similar to 31.6% (151/478) of patients who received PegIFNα-2b (*p* value = 0.1). Responses of HBsAg loss also showed no difference between the PegIFNα-2a and PegIFNα-2b arms (*p* value = 0.3). Neither host (gender, age, body mass index, weight, alanine transaminase) nor viral biomarkers (HBV RNA, HBV DNA, HBeAg, HBsAg, genotype) showed any significant difference between the PegIFNα-2a and PegIFNα-2b arms (Table S1). For subsequent analyses, patients who achieved serological responses and HBsAg loss were sorted into the SR and HL groups, respectively.

### Serum HBV RNA was strongly correlated with HBeAg

First, one-sample Kolmogorov–Smirnov tests showed that HBV RNA at individual sampling timepoints did not follow a normal distribution. Second, non-parametric Spearman correlation analyses revealed positive coefficients in the pairwise correlations between HBV RNA and HBeAg, HBsAg, HBV DNA. Third, HBV RNA was strongly correlated with HBeAg and HBV DNA (*p* values < 0.01), while its correlation coefficients with HBsAg were the weakest at week 0, 12, or 24 (Figure S2). Similar patterns were also observed in the PegIFNα-2a or PegIFNα-2b arm. Overall, strong correlations of HBV RNA with HBeAg supported that HBV RNA might be a potential predictor of HBeAg seroconversion.

### HBV RNA strongly predicted HBeAg seroconversion, but less so for HBsAg loss

**Fig. 2:**
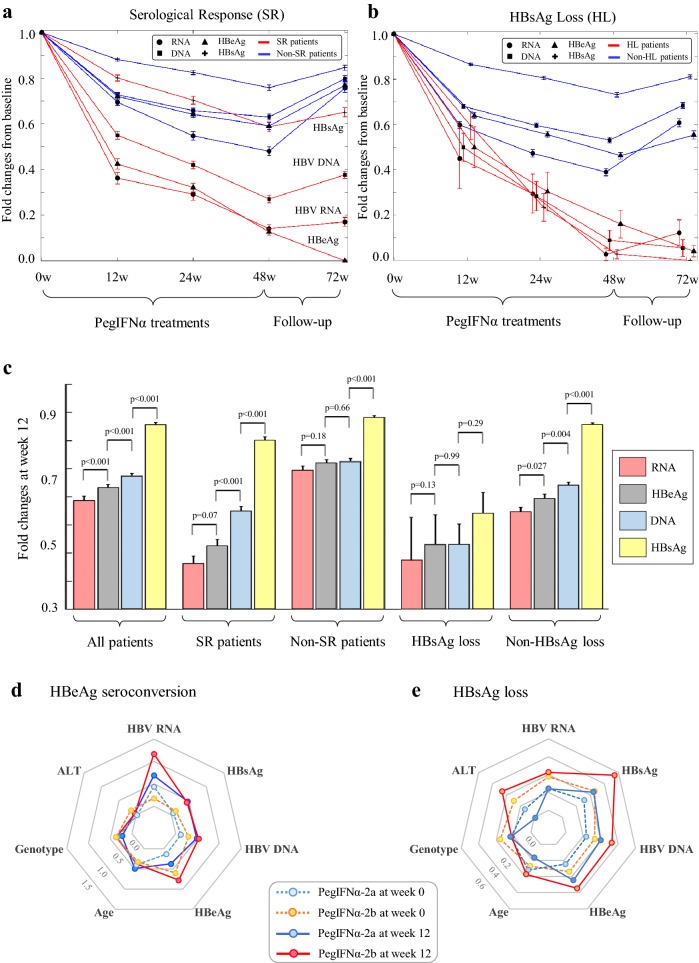
Fold changes and predictor importance estimates of HBV RNA, HBV DNA, HBsAg, and HBeAg. **a** Fold changes of HBV biomarkers in the SR (red lines) and non-SR patients (blue lines). **b** Fold changes of HBV biomarkers in HL and non-HL patients. **c** Comparisons of 12-week fold changes in five patient groups. Radar charts revealed predictor importance estimates of HBV biomarkers in the prediction of HBeAg seroconversion (**d**) or HBsAg loss (**e**). High values of predictor importance estimates indicate the significance of predictors

Three independent analyses were performed to explore if HBV RNA served as a favorable monitor to foretell interferon responses. First, HBV RNA shared a decreasing pattern similar to HBeAg in the SR group which harbored 217 patients with HBeAg seroconversion (Fig. 2a). HBV RNA decreased more rapidly in patients with HBsAg loss than patients without HBsAg loss (Fig. 2b). At week 12, a significant difference between HBV RNA decreases and HBeAg decreases was undetectable in the SR group (*p* value = 0.07), whereas pairwise comparisons between other factors all exhibited significant differences (*p* values < 0.001, Fig. 2c). Only 45 (6.2%) of 727 patients showed no decline of HBV RNA by week 12, while few of them achieved HBsAg loss (*n* = 1) or HBeAg seroconversion (*n* = 4). Two (9.5%) of 21 HL patients and 8 (3.7%) of 217 SR patients showed no decline of HBV RNA at week 12. Moreover, HBV RNA stabilization after treatment cessation from week 48 to 72 was more likely maintained in the SR than non-SR patients (*p* value < 0.01).

Second, univariate and multivariate regression analyses revealed HBV RNA as an effective predictor of HBeAg seroconversion (Table 2), despite its ineffectiveness for estimating HBsAg loss (Table S2). At weeks 0, 12, 24, and 48, univariate regression analyses consistently revealed that HBV RNA and other factors (HBV DNA, HBsAg, HBeAg, HBV genotypes, age, weight) were significantly associated with HBeAg seroconversion (*p* values < 0.05), whereas gender and PegIFNα types showed no difference. Multivariate analyses further showed that HBV RNA plus HBeAg and patient age remained significant at weeks 0, 12, 24, and 48 (Table 2). Regarding the prediction of HBsAg loss, HBV RNA at baseline and week 24 was a significant predictor in univariate analyses, but its significance disappeared in multivariate analyses probably due to the strong predictive role of HBsAg at all weeks (Table S2). Furthermore, predictor importance estimates independently revealed the significance of HBV RNA at week 12 in the SR prediction (Fig. 2d), whereas HBV RNA showed no advantage compared with HBsAg for estimating HBsAg loss (Fig. 2e).

**Table 2:** Logistic regression analyses of HBeAg seroconversion using the host and HBV biomarkers

Biomarkers	Univariate analyses		Multivariate analyses		Univariate analyses		Multivariate analyses	
	OR (95% CI)	*p* value	OR (95% CI)	*p* value	OR (95% CI)	*p* value	OR (95% CI)	*p* value
	**Week 0**				**Week 12**			
Age	0.95 (0.93–0.98)	1.9 × 10^−4^	0.94 (0.91–0.97)	3.3 × 10^−4^	0.95 (0.93–0.98)	1.9 × 10^−4^	0.93 (0.90–0.96)	1.6 × 10^−5^
Male gender	0.82 (0.58–1.16)	0.27			0.82 (0.58–1.16)	0.27		
Body weight	0.98 (0.96–0.99)	0.003			0.98 (0.97–0.99)	0.005		
PegIFNα-2a	0.78 (0.56–1.09)	0.15			0.78 (0.56–1.09)	0.15		
ALT	1.001 (1.0–1.002)	0.027	1.002 (1.0–1.003)	0.014	0.998 (0.996–1.0)	0.163		
Genotypes	0.50 (0.37–0.69)	1.7 × 10^−5^	0.49 (0.33–0.71)	1.8 × 10^−4^	0.50 (0.37–0.69)	1.7 × 10^−5^	0.63 (0.42–0.95)	0.026
HBV DNA	0.79 (0.64–0.97)	0.022			0.65 (0.60–0.72)	1.9 × 10^−19^		
HBsAg	0.55 (0.41–0.75)	1.3 × 10^−4^			0.42 (0.33–0.53)	7.4 × 10^−14^		
HBeAg	0.45 (0.34–0.6)	6.4 × 10^−8^	0.46 (0.31–0.67)	5.6 × 10^−5^	0.41 (0.35–0.49)	7.1 × 10^−25^	0.42 (0.31–0.58)	1.1 × 10^−7^
HBV RNA	0.75 (0.67–0.85)	3.16 × 10^−6^	0.79 (0.68–0.92)	0.002	0.43 (0.37–0.51)	1.2 × 10^−20^	0.52 (0.42–0.65)	4.1 × 10^−9^
	**Week 24**				**Week 48**			
Age	0.95 (0.93–0.98)	1.96 × 10^−4^	0.93 (0.89–0.96)	7.15 × 10^−5^	0.95 (0.93–0.98)	1.9 × 10^−4^	0.94 (0.91–0.97)	3 × 10^−4^
Male gender	0.82 (0.58–1.16)	0.27			0.82 (0.58–1.16)	0.27		
Body weight	0.98 (0.97–0.99)	0.015			0.98 (0.96–0.99)	0.012		
PegIFNα-2a	0.78 (0.56–1.09)	0.15			0.78 (0.56–1.09)	0.15		
ALT	.996 (.993–.999)	2.7 × 10^−3^			.995 (.992–.998)	0.007		
Genotypes	0.50 (0.37–0.69)	1.7 × 10^−5^			0.50 (0.37–0.69)	1.7 × 10^−5^	0.41 (0.61–0.91)	0.015
HBV DNA	0.63 (0.58–0.69)	4.32 × 10^−23^			0.52 (0.47–0.59)	0.001		
HBsAg	0.50 (0.42–0.60)	2.70 × 10^−13^			0.63 (0.56–0.71)	8.2 × 10^−14^	0.94 (0.91–0.97)	0.043
HBeAg	0.33 (0.27–0.40)	5.43 × 10^−29^	0.29 (0.19–0.43)	8.34 × 10^−10^	0.16 (0.12–0.22)	0.001	0.16 (0.11–0.23)	4.5 × 10^−24^
HBV RNA	0.38 (0.30–0.47)	4.23 × 10^−14^	0.64 (0.48–0.85)	0.0002	0.21 (0.14v0.32)	4.8 × 10^−14^	0.72 (0.61–0.88)	0.001

*CI* confidence interval, *OR* odds ratio

**Fig. 3:**
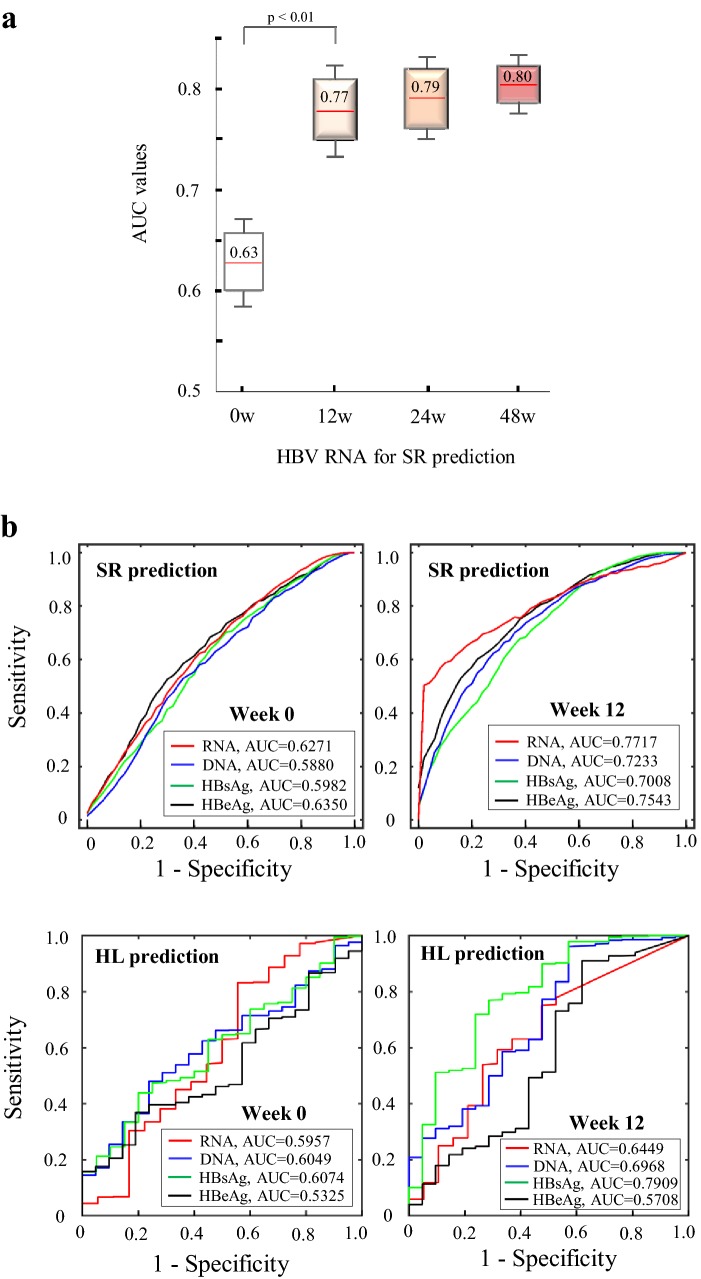
AUC performance of HBV biomarkers. **a** AUC values of HBV RNA at weeks 0, 12, 24, and 48. **b** Averaged ROC curves of four biomarkers at baseline and week 12. All data were used in the HL prediction without cross-validation due to the limited number of HL patients

Third, AUC analyses revealed the advantage of HBV RNA, especially at week 12, for predicting HBeAg seroconversion. In the SR prediction, AUC values of HBV RNA gradually increased from baseline (AUC = 0.63 ± 0.04) to week 12 (AUC = 0.77 ± 0.05), week 24 (AUC = 0.78 ± 0.04), and week 48 (AUC = 0.80 ± 0.03) (see Fig. 3a). A significant increase (ΔAUC = 0.14) was observed from baseline to week 12 (*p* value < 0.01). Moreover, HBV RNA showed an advantage over HBeAg (*p* value = 0.03) at week 12, and its performance was also superior to HBV DNA and HBsAg at weeks 0, 12, and 24 (*p* value < 0.05). However, compared with HBsAg, HBV RNA was only a modest predictor of HBsAg loss (Fig. 3b).

### Optimized cutoffs of HBV RNA improved the prediction of treatment responses

To identify simple cutoffs for clinical use, HBV biomarker cutoffs were optimized to forecast HBeAg seroconversion. The baseline cutoff of HBV RNA at 6.0 log_10_ copies/mL showed a modest performance of accuracy (61.6%) comparable to HBsAg and HBV DNA. Compared with baseline results, the optimized cutoff of HBV RNA ≤ 1000 copies/mL at week 12 offered significant increases of accuracy (76.6%), negative predictive value (85.3%), and positive predictive value (56.3%). The former two measures showed the best performance compared with HBeAg, HBsAg, and HBV DNA cutoffs (Table S3).

A negative predictive value above 95% is a useful indication to discontinue interferon treatments. At week 12, HBV RNA > 5.2 log_10_ copies/mL was observed in 174 (95.6%) of 182 patients who failed to achieve HBeAg seroconversion. This cutoff also showed the best performance of accuracy and positive predictive value compared to that of HBV DNA, HBsAg, and HBeAg cutoffs at week 12 (Table S3).

### HBV RNA plus traditional markers improved the estimation of interferon responses

**Fig. 4:**
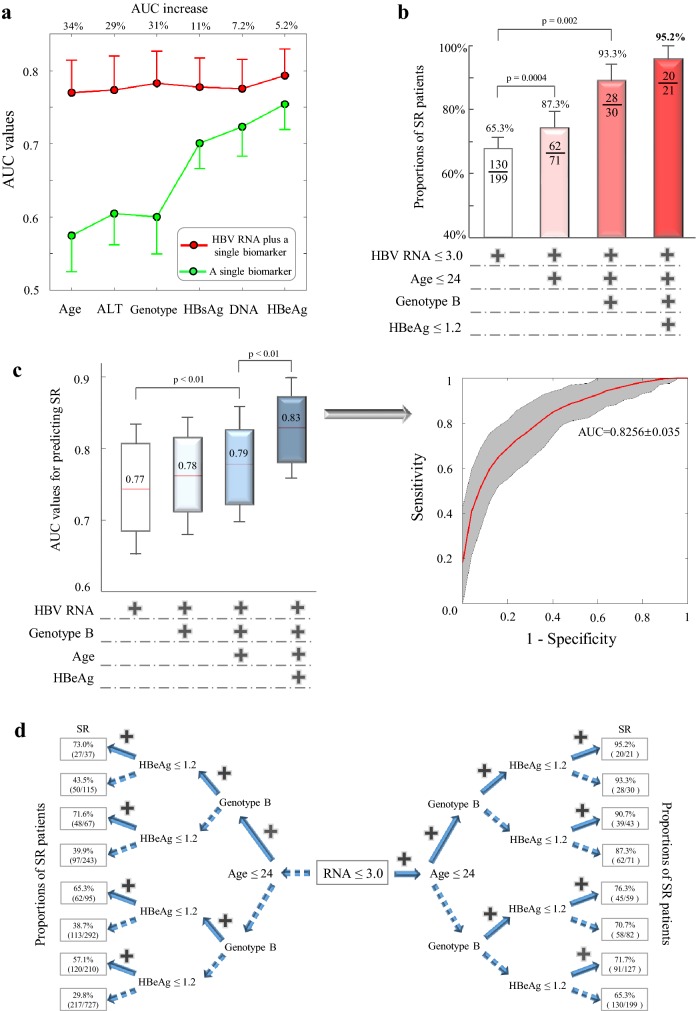
Predictive performance of biomarker cutoffs and their combined performance. **a** AUC performance of dual combinations containing HBV RNA plus single conventional biomarkers. AUC values of dual combinations were significantly higher compared to that of individual biomarkers (*p* values < 0.01, see increased percentages on top). **b** Proportions of SR patients incrementally stratified by four 12-week biomarkers. **c** AUC values of 12-week biomarker combinations in the SR prediction. The average ROC curve on right was indicated by the red line and gray area showed the 95% confidence interval. **d** Proportions of SR patients in combinations of four 12-week biomarkers (HBV RNA, genotype B, HBeAg, age). A solid branch indicates the inclusion of a biomarker, while a dotted branch indicates that a biomarker was not used in the calculation. For instance, 65.3% (130/199) at the bottom right was the SR proportion under the single condition of HBV RNA ≤ 3 log_10_ copies/mL, while 95.2% (20/21) at the top left was the SR proportion with four conditions fulfilled simultaneously

It has been hypothesized that multiple factors indicated by multivariate regression analyses may increase predictive performance albeit interferons only offer 18–39% of HBeAg seroconversion in clinical studies (Table S4). This hypothesis was supported by dual combinations that adding HBV RNA to individual traditional HBV biomarkers significantly increased AUC performance (*p* value < 0.01) (Fig. 4a).

We next screened all 127 combinations of 7 available factors (patient age, ALT, HBV genotype, HBV RNA, HBV DNA, HBsAg, HBeAg) to identify the best combination that achieved the highest rate of SR with the minimum variable size. A combination of four 12-week factors (HBV RNA ≤ 3.0 log_10_ copies/mL, patient age ≤ 24, genotype B, HBeAg ≤ 1.2 log_10_ COI) offered the best performance that their stepwise combinations gradually increased the accuracy (95.2%) to predict HBeAg seroconversion (Fig. 4b). In the stepwise combinations of HBV RNA plus genotype B, patient age, and HBeAg, their AUC values significantly increased from 0.77 to 0.83 (*p* value < 0.01, Fig. 4c). As illustrated in a decision tree model (Fig. 4d), this four-factor combination offered superior performance (accuracy 95.2%, true negative rate: 99.8%) to forecast HBeAg seroconversion even at the early period of week 12.

## Discussion

This study reveals the significant role of serum HBV RNA to forecast treatment responses of PegIFNα-2a and PegIFNα-2b in 727 HBeAg-positive patients, which is the largest cohort study of HBV RNA reported so far. Our study reveals three major findings that support the use of HBV RNA as an early on-treatment factor to estimate PegIFNα responses. First, HBV RNA is not only positively correlated with traditional HBV biomarkers but also acts as a strong predictor of HBeAg seroconversion and a modest predictor of HBsAg loss. Second, HBV RNA at week 12 is an early predictor and its optimized cutoff at approximately 1000 copies/mL effectively forecasts HBeAg seroconversion, supporting the hypothesis that the suppression of HBV RNA at a low level may lead to better treatment outcomes. Third, HBV RNA plus traditional factors such as patient age, HBV genotype, and HBeAg considerably forecast PegIFNα responses, even at the early period of week 12. An early prediction of treatment responses is of great clinical value, because PegIFNα treatments are usually expensive and cause severe adverse events in the long term [22].

Serum HBV RNA was previously reported as a potential biomarker to estimate treatment outcomes of HBV polymerase inhibitors [11, 12, 23, 24] or PegIFNα-2a in a small cohort of 76 responder-enriched patients [13]. In our study, HBV RNA was evaluated using a randomized phase 3 cohort of 727 HBeAg-positive patients. Our univariate and multivariate regression analyses consistently revealed the predictive value of HBV RNA in the SR prediction at weeks 0, 12, 24, and 48 (Table 2). Moreover, we observed that HBV RNA was positively correlated with HBeAg, HBV DNA, and HBsAg at all sampling points, which was in agreement with the literature results [25]. HBV RNA decreased faster than HBV DNA and HBsAg at the early treatment period, whereas its decreasing pattern in SR patients was similar to HBeAg, supporting its potential to predict HBeAg seroconversion (Fig. 2). In contrast, HBV RNA was only a modest predictor of HBsAg loss in our analyses. This was in agreement with a recent review which highlighted that HBsAg was a key predictor of HBsAg loss, but other biomarkers seemed less effective [26].

Kinetics of HBV RNA may vary due to different therapies and treatment duration, but a decline of serum HBV RNA is commonly observed during the treatment of interferons and/or nucleos(t)ide analogs [12, 13, 23]. In agreement with previous findings on nucleos(t)ide analogs [12], our study revealed the effectiveness of HBV RNA at week 12 to predict HBeAg seroconversion for interferon treatments. By monitoring HBV RNA dynamics over 72 weeks, we found that fast decreasing HBV RNA at week 12, but not the baseline HBV RNA, was a sound predictor of HBeAg seroconversion. A recent study, however, reported that HBV RNA outperformed HBeAg at baseline in 76 response-enriched patients treated with PegIFNα-2a [13]. This disagreement might be due to their cohort which only included response-enriched patients (*n* = 76, SR = 51%) screened from two different clinical trials (*n* = 271, SR = 32.1% and *n* = 130, SR = 36.2%). Despite this, our results and literature results support that low levels and fast decreases of HBV RNA are likely associated with treatment success even in the early period [23, 24].

HBV biomarker cutoffs are commonly used to monitor treatment responses in clinical practice. Based on our cohort of 727 patients, the 12-week cutoff of HBV RNA was optimized at 1000 copies/mL that effectively predicted HBeAg seroconversion. The favorable performance of HBV RNA cutoff was also highlighted by its better accuracy (76.6%) compared with HBV DNA, HBsAg, and HBeAg cutoffs (Table S3). Furthermore, early discontinuation of interferon treatments could be considered in HBeAg-positive patients who showed HBV RNA > 5.2 log_10_ copies/mL at week 12, because more than 95% of these patients were unable to achieve HBeAg seroconversion. In addition to the predictive performance of individual biomarkers, our study proved that multivariate combinations of HBV RNA plus HBeAg, patient age, and genotype B offered high levels of positive predictive value (95.2%) and specificity (99.8%). This provides clear evidence that HBV RNA can be effectively integrated with traditional biomarkers to monitor treatment responses.

There are limitations to our study. We analyzed a phase 3 clinical trials of 727 Chinese patients mainly infected with HBV genotype B or C (98.9%), while future studies need to recruit patients infected with other HBV genotypes. Our study focused on interferon treatments in HBeAg-positive non-cirrhotic patients, while future analyses need to clarify HBV RNA in HBeAg-negative patients and associations between HBV RNA and cirrhosis or hepatocellular carcinoma. The full-length HBV RNA could be quantified using our primers taken from previous publications [12, 23], but further studies need to confirm whether the encapsulated, polyadenylated serum HBV RNA is equal to full-length RNA or contains truncated RNA species [7]. Due to limited patients with HBsAg loss (2.9%) in our 72-week cohort, the associations of HBV RNA with HBsAg loss will be evaluated by our 5-year follow-up study.

Overall, our findings suggest that serum HBV RNA may serve as an early on-treatment predictor of HBV cccDNA activity to reveal the treatment success of interferon therapies. Although standard HBV RNA toolkits are yet to be developed for clinical use [7], the preliminary clinical significance of HBV RNA is supported by our large-scale study. The discovery of HBV RNA as an effective and early biomarker may lead to better management of interferon therapies for HBV-infected patients, driving the clinical use of HBV RNA [11, 12, 27].

## Electronic supplementary material

Below is the link to the electronic supplementary material.

Supplementary material 1 (PDF 1512 kb)

## Abbreviations

ALT: Alanine aminotransferase
anti-HBe: Hepatitis B e antibody
anti-HBs: Hepatitis B s antibody
AUC: Area under the receiver operating characteristic curve
cccDNA: Covalently closed circular DNA
CI: Confidence interval
COI: Cut of index
CR: Combined response
HBeAg: Hepatitis B e antigen
HBsAg: Hepatitis B s antigen
HL: HBsAg loss
NA: Nucleos(t)ide analog
NPV: Negative predictive value
OR: Odds ratio
PegIFNα-2a: Pegylated interferon alfa-2a
PegIFNα-2b: Pegylated interferon alfa-2b
PPV: Positive predictive value
ROC: Receiver operating characteristic curve
SR: Serological response
