# Supplementary material for: Rapidly decreased HBV RNA predicts responses of pegylated interferons in HBeAg-positive patients: a longitudinal cohort study
Source: Hepatol Int. 2020 Feb 25;14(2):212–24. doi: 10.1007/s12072-020-10015-3 (PMC7136184; doi:10.1007/s12072-020-10015-3)
Supplement: Supplementary file 1 — Supplementary material 1 (PDF 1512 kb) [file 12072_2020_10015_MOESM1_ESM.pdf]

Supplementary data

Rapidly decreased HBV RNA predicts responses of pegylated interferons in HBeAg-positive patients: a longitudinal cohort study

Min Zhang[#], Guangdi Li[#], Jia Shang, Chen Pan, Minxiang Zhang, Zhibiao Yin, Qing Xie, Yanzhong Peng, Qing Mao, Xinqiang Xiao, Yongfang Jiang, Kaizhong Luo, Yun Xu, Hai Ding, Wenzhou Fan, Vidaurre Diego, Mahmoud Reza Pourkarim, Erik De Clercq, Guiqiang Wang, Guozhong Gong

Contents

Supplementary Figures 2

Supplementary Tables.. 4

Supplementary Methods 8

References 20

Supplementary Figures

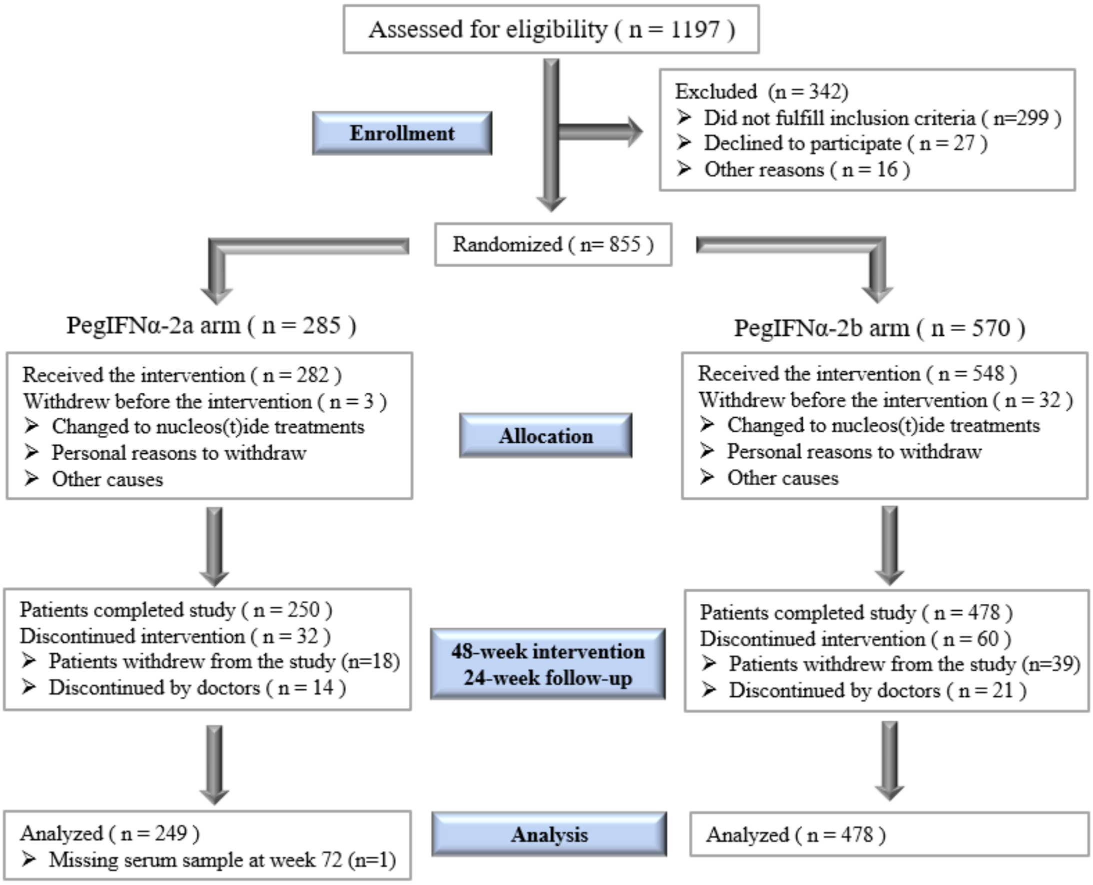

Figure S 1. Patient disposition throughout this study. This figure was adapted from the phase 3 clinical study which was previously published in Chinese [1].

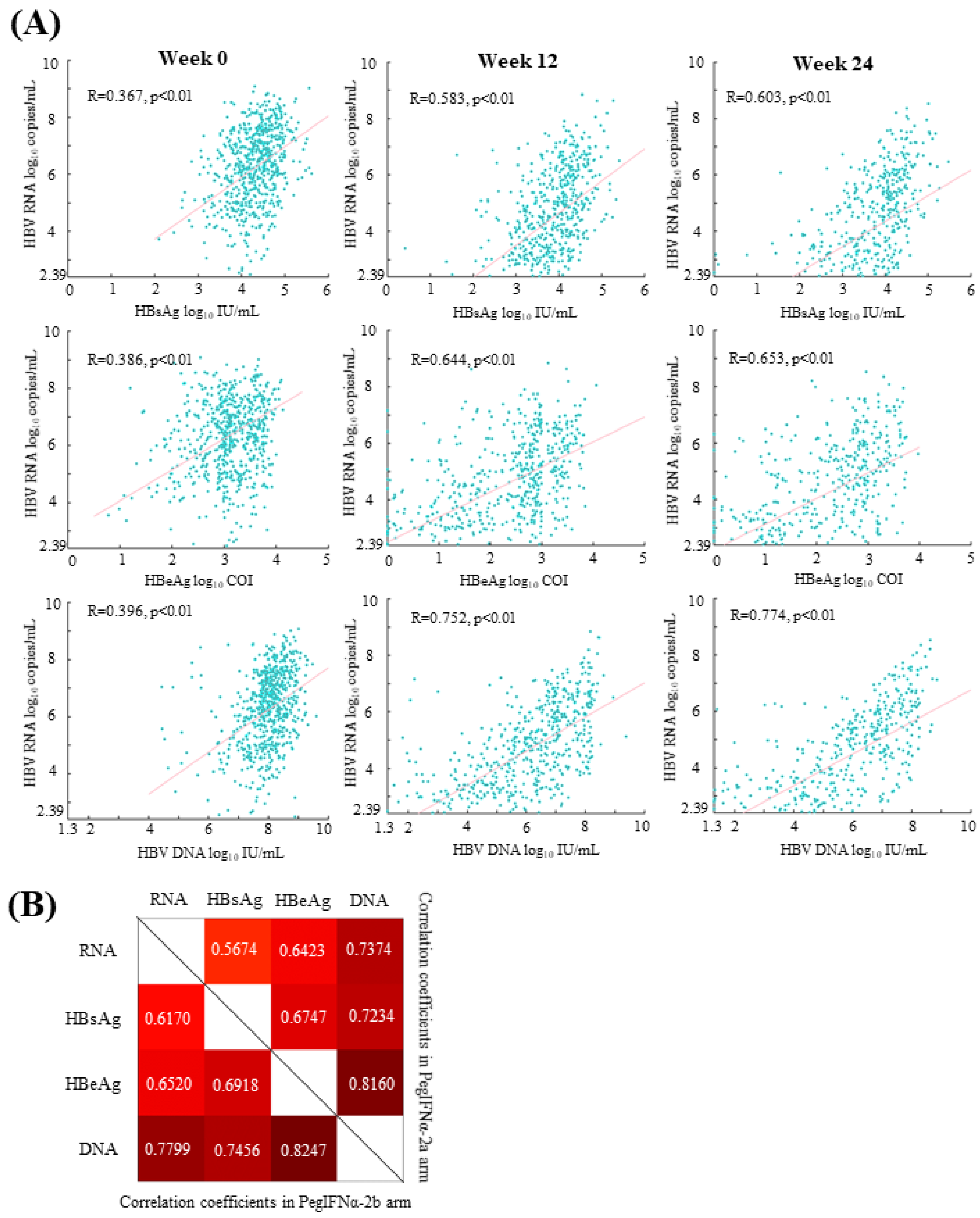

Figure S 2. Pairwise correlation plots of HBV RNA correlated with HBV DNA, HBsAg, and HBeAg based on our cohort of 727 patients.

(A) HBV RNA was positively correlated with HBeAg, HBsAg, and HBV DNA at weeks 0, 12, and 24. Spearman correlation coefficients and their statistical significance are indicated in the subfigures.

(B) Correlation matrix showing correlation coefficients between four 12-week HBV biomarkers in the arms of PegIFNα-2a (top right) and PegIFNα-2b (bottom left).

Supplementary Tables

Table S 1. Characteristics of biomarkers in HBeAg-positive patients from the PegIFNα-2a and PegIFNα-2b arms.

**Biomarkers**	**PegIFNα-2b (n=478)**	**PegIFNα-2a (n=249)**	**p-value***
**Gender**			
Male	346 (72.4%)	177 (71.1%)	0.71
Female	132 (27.6%)	72 (28.9%)	0.71
**Age**	28.21 ± 0.32	28.09 ± 0.41	0.93
**BMI**	22.15 ± 0.13	21.92 ± 0.18	0.27
**Weight**	63.95 ± 0.53	62.82 ± 0.69	0.30
**ALT**	180.43 ± 4.08	181.64 ± 4.90	0.21
**HBV genotypes**			0.91
B	195 (40.8%)	97 (38.9%)	0.63
C	277 (56.0%)	150 (60.2%)	0.55
D	5 (1.05%)	2 (0.8%)	0.75
B+C	1 (0.21%)	0 (0%)	0.47
**HBV RNA**#			
0w	6.17 ± 0.07	6.43 ± 0.08	0.07
12w	4.05 ± 0.07	4.30 ± 0.11	0.51
24w	3.64 ± 0.07	4.19 ± 0.12	0.12
48w	3.49 ± 0.07	3.65 ± 0.11	0.10
72w	4.37 ± 0.09	4.52 ± 0.13	0.47
**HBV DNA**#			
0w	7.94 ± 0.03	7.93 ± 0.05	0.95
12w	5.38 ± 0.09	5.34 ± 0.12	0.71
24w	4.70 ± 0.10	4.76 ± 0.14	0.71
48w	4.36 ± 0.11	4.19 ± 0.15	0.58
72w	5.40 ± 0.12	5.47 ± 0.17	0.68
**HBsAg**#			
0w	4.28 ± 0.02	4.28 ± 0.03	0.78
12w	3.66 ± 0.04	3.65 ± 0.05	0.65
24w	3.32 ± 0.05	3.39 ± 0.06	0.50
48w	2.95 ± 0.07	2.99 ± 0.08	0.55
72w	3.29 ± 0.06	3.34 ± 0.07	0.9994
**HBeAg**#			
0w	3.03 ± 0.03	3.03 ± 0.04	0.88
12w	1.96 ± 0.05	1.98 ± 0.07	0.95
24w	1.69 ± 0.05	1.68 ± 0.07	0.91
48w	1.46 ± 0.06	1.34 ± 0.08	0.38
72w	1.65 ± 0.07	1.72 ± 0.10	0.40

**HBeAg seroconversion**	151 (31.6%)	66 (26.5%)	0.155
**HBsAg loss (HI)**	16 (3.4%)	5 (2.0%)	0.31

*: p-values were measured by nonparametric Mann–Whitney U tests to show the difference in HBeAg-positive patients receiving either PegIFNα-2a or PegIFNα-2b during the 48-week course of interferon treatment and the 24-week treatment-free follow-up.

#: HBV RNA: log_{10} copies/mL, HBV DNA: log_{10} IU/mL, HBsAg: log_{10} IU/mL, HBeAg: log_{10} COI.

&: Mean and standard error were listed.

Table S 2. Logistic regression analyses of host and HBV biomarkers in the prediction of HBsAg loss.

	**Week 0**				**Week 12**			
	Univariate Analyses		Multivariate Analyses		Univariate Analyses		Multivariate Analyses	
Biomarkers	OR (95% CI)	p-value	OR (95% CI)	p-value	OR (95% CI)	p-value	OR (95% CI)	p-value
Age	0.99 (0.92 ~ 1.05)	0.71			0.99 (0.92 ~ 1.05)	0.71		
Male gender	0.42 (0.17 ~ 0.99)	0.049			0.42 (0.17 ~ 0.99)	0.049		
PegIFNα-2a	0.59 (0.21 ~ 1.63)	0.31			0.59 (0.21 ~ 1.63)	0.31		
Genotypes	0.49 (0.21 ~ 1.16)	0.10			0.49 (0.21 ~ 1.16)	0.10		
Body weight	0.95 (0.90 ~ 0.99)	0.02	0.94(0.9~0.98)	0.005	0.94 (0.90 ~ 0.99)	0.016		
ALT	1.00(0.997~1.003)	0.91			1.003(1.0 ~ 1.006)	0.034	1.004(1.0~1.007)	0.043
HBV DNA	0.75 (0.46 ~ 1.22)	0.24			0.68 (0.53 ~ 0.86)	0.001		
HBsAg	0.47 (0.23 ~ 0.99)	0.047	0.28 (0.14~0.54)	1.4×10^{-4}	0.23 (0.14 ~ 0.37)	2.3×10^{-9}	0.17 (0.09~0.32)	2.6×10^{-8}
HBeAg	1.42 (0.63 ~ 3.21)	0.04	3.41(1.24~9.4)	0.018	0.76 (0.51 ~ 1.11)	0.15	2.2 (1.17 ~ 4.2)	0.015
**HBV RNA**	**0.72 (0.54 ~ 0.97)**	**0.03**			**0.71 (0.51~1.004)**	**0.053**		
	**Week 24**				**Week 48**			
Age	0.99 (0.92 ~ 1.05)	0.71			0.99 (0.92 ~ 1.05)	0.71	1.22(1.04~1.42)	0.016
Male gender	0.42 (0.17 ~ 0.99)	0.049			0.42 (0.17 ~ 0.99)	0.049		
PegIFNα-2a	0.59 (0.21 ~ 1.63)	0.31			0.59 (0.21 ~ 1.63)	0.31		
Genotypes	0.49 (0.21 ~ 1.16)	0.10			0.49 (0.21 ~ 1.16)	0.10		
Body weight	0.94 (0.89 ~ 0.98)	0.01	0.94(0.9~0.98)	0.005	0.94 (0.90 ~ 0.99)	0.026		
ALT	.998(0.99~1.005)	0.54			0.99(0.978~1.004)	0.178		
HBV DNA	0.55 (0.41 ~ 0.75)	0.14			0.19 (0.06 ~ 0.61)	0.005		
HBsAg	0.18 (0.11 ~ 0.29)	3.95×10^{-13}	0.28 (0.14~0.54)	1.4×10^{-4}	0.06 (0.02 ~ 0.18)	1.5×10^{-7}	.028 (.006 ~ 0.13)	7×10^{-6}
HBeAg	0.50 (0.31 ~ 0.80)	0.004	3.41(1.24~9.4)	0.018	0.42 (0.24 ~ 0.74)	0.0027		
**HBV RNA**	**0.58 (0.36 ~ 0.95)**	**0.03**			**0.14 (0.017~1.09)**	**0.06**		

Table S 3. Early prediction of HBeAg seroconversion by HBV biomarker cutoffs.

	Cutoff	Sensitivity	Specificity	PPV	NPV	Accuracy	AUC
**Week 0**							
HBV RNA	≤ 6.0	50.8%	65.8%	37.6%	76.7%	61.6%	0.63
	> 8.2	99.5%	6.3%	30.1%	96.8%	33.2%	
HBV DNA	≤ 7.9	50.7%	66.3%	39.0%	76.0%	61.5%	0.59
	> 9.2	100%	2.2%	30.3%	100%	31.4%	
HBsAg	≤ 4.3	60.2%	57.5%	36.0%	75.5%	57.1%	0.60
	> 5.0	100%	6.5%	31.3%	100%	34.4%	
HBeAg	≤ 2.9	57.6%	65.3%	41.4%	78.4%	62.5%	0.64
	> 3.9	99.5%	3.7%	30.6%	95%	32.3%	
**Week 12**							
HBV RNA	≤ 3.0	66.0%	79.4%	56.3%	85.3%	76.6%	0.77
	> 5.2	95.9%	35.4%	37.4%	95.6%	52.8%	
HBV DNA	≤ 4.5	55.8%	75.3%	48.8%	80.1%	69.5%	0.72
	> 8.1	99.1%	7.9%	31.2%	95.2%	34.9%	
HBsAg	≤ 3.4	47.4%	72.9%	42.5%	76.7%	65.3%	0.70
	> 4.4	97.7%	18.9%	33.7%	95.1%	42.3%	
HBeAg	≤ 1.2	53.0%	84.1%	58.7%	80.8%	74.8%	0.75
	> 3.3	98.2%	15.1%	33.0%	95.1%	39.9%	

PPV: positive predictive value (also known as precision), NPV: negative predictive value. Accuracy is defined by the proportion of true positives plus true negatives among all cases. Biomarker units are measured by $\log_{10}$ copies/mL for HBV RNA, $\log_{10}$ IU/mL for HBV DNA, $\log_{10}$ IU/mL for HBsAg, and $\log_{10}$ COI for HBeAg.

Table S 4. Summary of HBeAg seroconversion and HBsAg loss in HBeAg-positive patients treated with PegIFNα-2a or PegIFNα-2b.

Study design	HBV genotypes	HBeAg seroconversion % (n)	HBsAg loss % (n)	Ref.
**Peginterferon α-2a treatment**				
Peginterferon α-2a 180μg for 48 weeks plus a treatment-free follow-up for 24 weeks.	B, C	39.1% (52/133)	2.2% (3/133)	[2]
Peginterferon α-2a 180μg for 48 weeks plus a treatment-free follow-up for 24 weeks.	A, B, C, D	36.15% (47/130)	2.3% (3/130)	[3]
Peginterferon α-2a 180μg for 48 weeks plus a treatment-free follow-up for 24 weeks.	A, B, C, D	32.1% (87/271)	NA	[4]
Peginterferon α-2a 180μg for 24 weeks plus a treatment-free follow-up for 24 weeks.	B, C	32.61% (15/46)	NA	[5]
Peginterferon α-2a 180μg for 48 weeks plus a treatment-free follow-up for 24 weeks.	B, C	32.02% (73/228)	NA	[6]
**Peginterferon α-2b treatment**				
Peginterferon α-2b 100μg for 52 weeks plus a treatment-free follow-up for 26 weeks.	A, B, C, D	18.02% (20/111)	0.9% (1/111)	[7]
Peginterferon α-2b 100μg for 52 weeks plus a treatment-free follow-up for 26 weeks.	A, B, C, D	28.68% (39/136)	6.6% (9/136)	[8]
Peginterferon α-2b 100μg for 52 weeks plus a treatment-free follow-up for 26 weeks.	A, B, C, D	30.81% (53/172)	6.9% (12/172)	[9]
**Nucleos(t)ide analogue treatment**				
Two reviews summarized the efficacy of nucleos(t)ide analogues in clinical trials.		16% to 21% for lamivudine, 21% for entecavir, 12% for adefovir, 22% for telbivudine, 21% for tenofovir	<1% for lamivudine, 2% for entecavir, 0% for adefovir, telbivudine, 3% for tenofovir	[10, 11]

Supplementary Methods

In this section, we provide details about HBV RNA extraction and quantification.

Primers, probes, and internal controls

In order to measure serum HBV RNA, primers of 3'RACE, HBV RNA-forward, HBV RNA-reverse, and HBV RNA-probes were obtained in literature [12]. Noncompetitive internal controls were added to monitor the extraction and amplification of viral loads [13]. Information on primers, probes, and internal controls is summarized below.

Table S 5. Oligonucleotide sequences of primers and probes for HBV RNA quantification.

Primers and probes	Nucleotide sequences (5' to 3')	NT position
HBV RNA-RACE*	ACC ACG CTA TCG CTA CTC AC $(dT)_{17}$GWA GCT C	1935-1929
HBV RNA-forward*	ACC ACG CTA TCG CTA CTC AC	
HBV RNA-reverse*	CAA CTT TTT CAC CTC TGC CTA	1817-1837
HBV RNA-probe*	FAM-CAT GTC CYA CTG TTC AAG CCT CCA AG-BHQ1	1851-1876
Internal control (IC)	GTC CAG TAC TTT CAA AGC TCG ATC CCG GTA ACT ACC AAA TCG GTA CGT ACC GGT TTA AAA CCA CCG ATC GCC TCT TCC CAA CCT GTA CGT ACG TAC GTA CGT CCA AAA GTT TCC ACG TAC GAT CGA TC	
Internal control forward	GCT CGA TCC CGG TAA CTA CCA	
Internal control reverse	GTA CGT GGA AAC TTT TGG ACG	
Internal control probe	HEX- TCG GTA CGT ACC GGT TTA AAA CCA CC-BHQ1	

*: Primers of RNA-RACE, HBV RNA-forward, HBV RNA-reverse, and HBV RNA-probe were obtained in the literature [12]. HBV RNA-RACE primer (ACC ACG CTA TCG CTA CTC AC $(dT)_{17}$GWA GCT C) was used to amplify polyadenylated HBV RNA without DNA degradation [12].

Here, we collected HBV genomic sequences to prove that primer sequences above were located within the conserved regions of the HBV genome. First, fifteen HBV genomic sequences were extracted from the NCBI nucleotide database (https://www.ncbi.nlm.nih.gov/nuccore/?term=HBV). Second, the NCBI genotyping

tool (http://www.ncbi.nlm.nih.gov/projects/genotyping/formpage.cgi) was used to assign HBV genotype classifications. Third, a consensus sequence was generated in the primer regions of the HBV genome.

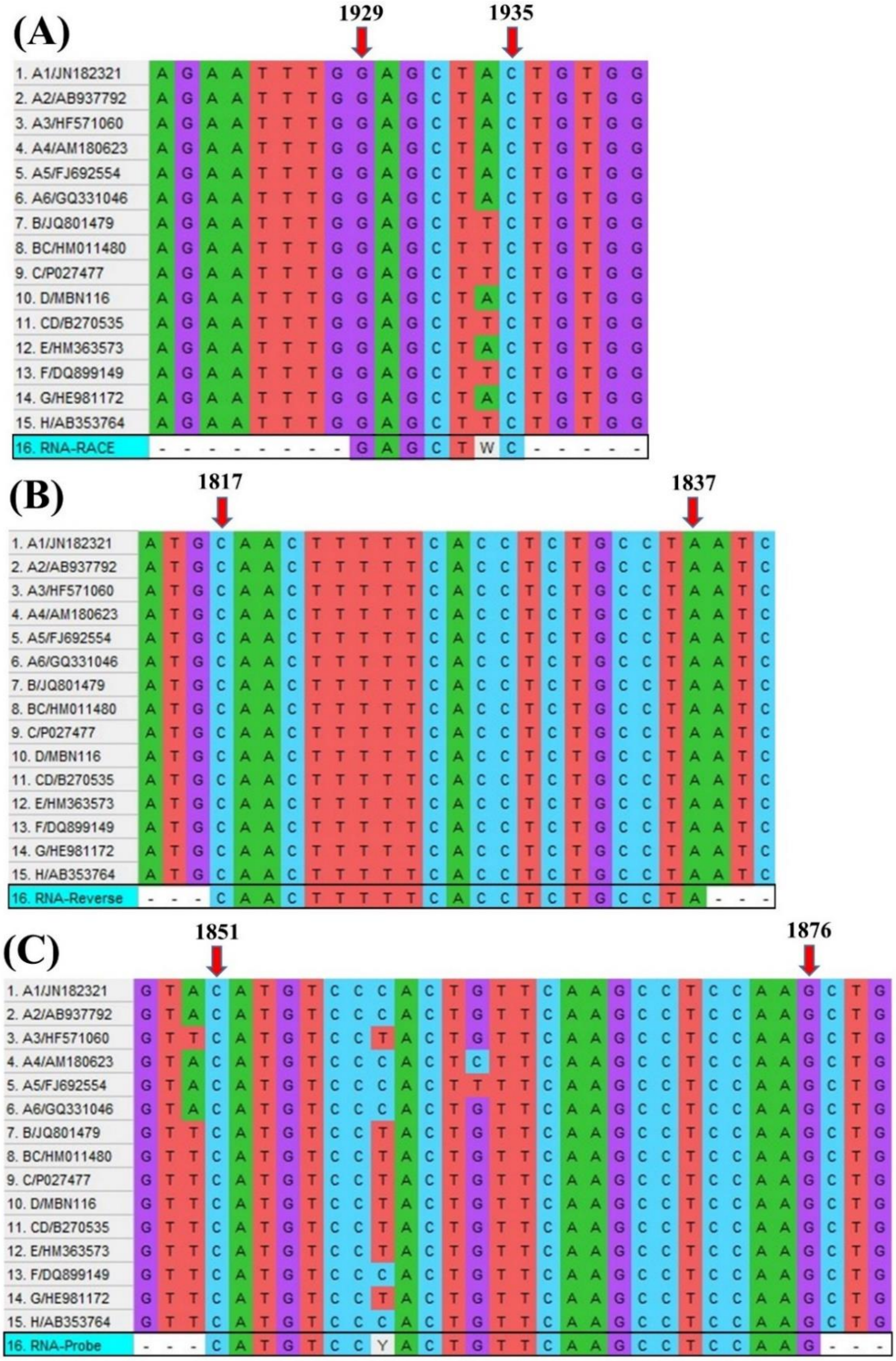

Figure S 3. **Conservation of primer sequences for HBV RNA detection.** From

subfigures (A) to (C), three primer sequences (RNA-RACE, HBV-reverse, HBV-Probe) were aligned individually with 15 sequences whose HBV genotypes were classified as A1 (sequence ID: JN182321), A2 (AB937792), A3 (HF571060), A4 (AM180623), A5 (FJ692554), A6 (GQ331046), B (JQ801479), C (P027477), D (MBN116), E (HM363573), F (DQ899149), G (HE981172), H (AB353764) plus two recombinant forms BC (HM011480) and CD (B270535). For each primer, its nucleotide positions indexed in the full-length HBV genome are shown on top of multiple sequence alignments. Primer sequences were the consensus of different HBV genotype sequences, and ambiguous nucleotides were taken into account (e.g. "W" represents "A" or "T"; "Y" represents "C" or "T"). MEGA X was used for data visualization.

Three subfigures above highlight the conserved regions of primers. In these figures, three primer sequences (RNA-RACE, HBV-reverse, HBV-Probe) were individually aligned with 15 sequences whose HBV genotypes were classified as A1, A2, A3, A4, A5, A6, B, C, D, E, F, G, H plus two recombinant forms BC and CD. Above findings suggest that primer sequences are conserved across different HBV genotypes. Taken together, above primer sequences within conserved regions could be potentially used to detect HBV RNA in different HBV genotypes.

HBV RNA extraction and amplification

HBV RNA extraction, PCR reaction, and amplification were performed on the Natch S system (Sansure Biotech, Inc, China) using protocols, reagents, and software provided by the manufacturer [14]. HBV RNA was extracted from patient serum samples (200 µL) using a nucleic acid extraction kit which was developed based on the magnetic bead technology. According to the manufacturer's protocol, HBV RNA extraction could be briefly described by seven-fold processes.

(1) Prepare an appropriate number of centrifugation tubes (1.5 mL). Mark negative control, positive control, and serum sample names on tubes accordingly. Add 600

μL of RNA Extraction Solution 1 into each tube. Note that RNA Extraction Solution 1 to 4 are available in the nucleic acid extraction kit.

(2) Add serum sample (200 μL), negative control, and positive internal control into each centrifugation tube. Close the lip, vortex it for 10 seconds, then centrifuge them instantaneously.

(3) Add 100 μL of RNA Extraction Solution 2 into each tube (mix it thoroughly before pipetting), vortex it for 10 seconds, then keep it at the temperature of 60℃ for 10 minutes, followed by 4℃ for 10 minutes.

(4) Centrifuge them instantaneously and then put centrifugation tubes in a separator. After 3 minutes, pipette the liquids out slowly (do not touch the brown substance on the tube wall).

(5) Add 600 μL of RNA Extraction Solution 3 and 200 μL of RNA Extraction Solution 4 into each tube, and vortex it for 5 seconds. Centrifuge tubes instantaneously and then put them into a separator again.

(6) After three minutes, place the pipette tip at the bottom of centrifugation tubes and slowly pipette all liquids inside. Hold it for one minute, then pipette the residual liquid at the bottom of the tube.

(7) Add 30 μL of elution buffer to elute magnetic beads on the tube wall until all magnetic beads are removed to the bottom of tubes. Pipette it for 3 or 4 times and then hold the tube at the room temperature for 10 minutes. Afterward, place centrifugation tubes in a separator for 3 minutes, then transfer this eluted RNA to another centrifugation tube (1.5 mL).

Notably, all centrifugation tubes, pipettes, PCR tubes, and related equipment used above should be RNase free to prevent the interference of RNase.

Eluted HBV RNA (30 μL) was obtained from HBV RNA extraction above and 10 μL was used for reverse transcription. Real-time fluorescence quantitative PCR technology was applied to quantify HBV RNA using fluorescence probes and specific

primers that target conserved HBV regions (see **Table S 5**). In one run, a total of 96 samples (16 samples/batch × 6 batches) including 90 patient samples, 4 RNA standards (concentrations: 5×10^5, 5×10^6, 5×10^7, 5×10^8 copies/mL), positive control, and negative control were prepared in 96 tubes. For each tube, 10 μL of the eluted RNA (obtained from HBV RNA extraction) plus necessary reagents (e.g. primers, reverse transcriptase, buffer solution, dNTPs, cations) were added. In the absence of DNA polymerase and cDNA primers, HBV RNA was reverse transcribed into complementary DNA (cDNA) under the temperature of 50℃ for 30 minutes during the reverse transcription. These cDNAs were produced with a specific anchor (5'-ACCACGCTATCGCTACTCAC-3') from the HBV RNA-RACE primer. During the amplification process, DNA polymerase and cDNA primers were added into tubes, whereas the reverse transcriptase was inactivated. Amplification was performed by an activation step at 95°C for 2 minutes, followed by 50 two-step cycles (each cycle: 15 seconds at 95 ℃ and 30 seconds at 60°C), and a cooling step down to 25°C for 10 seconds. The fluorescence of cDNA in the tubes was detected and measured by the 7500 Real-Time PCR System (Applied Biosystems®).

Internal control

To provide effective internal control, armored RNA technology was applied to encode predefined RNA sequences (see internal control in **Table S 5**) in noninfectious pseudoviral particles protected from plasma ribonucleases (see more details in literature [13, 15-17]). Note that the armored RNA is widely applied as external and internal controls in nucleic acid-based assays for the quantification of viral loads in clinical samples [13]. Internal control sequences should exhibit normal amplification curves with expected Ct values; otherwise, problem shooting will be conducted to seek any potential mistake.

HBV RNA standard

Stable standards are required for quantifying HBV RNA levels in patient serums. We prepared HBV RNA standards similar to the approach proposed in the literature

[18]. Here, we briefly describe the preparation of HBV RNA standards in threefold procedures. First, a plasmid pGS-1 ligated with a replication sequence was constructed: ATGCAACTTTTTCACCTCTGCCTAATCATCTCTTGTTCATGTCCTACTGTTCA AGCCTCCAAGCTGTGCCTTGGGTGGCTTTGGGGCATGGACATTGACCCTTA TAAAGAATTTGGAGCTTCAAAAAAAAAAAAAAAAAGTGAGTAGCGATAGC GTGGT. Second, the constructed plasmid was quantified by a spectrophotometer (Thermo Scientific™ NanoDrop 2000). Third, Tris-EDTA buffer solution (pH=8.0) was added to dilute quantitative references with HBV RNA concentrations ranged from 5×10^2 to 5×10^9 copies/mL.

HBV RNA quantification in the presence of HBV DNA

Below, we show that HBV RNA detection is unlikely influenced even if serum samples were pretreated by deoxyribonuclease I (DNase I). Experiments were designed to prove this result under four conditions:

(a) HBV RNA negative and DNA negative;

(b) HBV RNA negative and DNA positive;

(c) HBV RNA positive and DNA negative;

(d) HBV RNA positive and DNA positive.

First, we conducted experiments to validate the HBV RNA detection system under the first three conditions: (a) TE buffer (pH=8.0); (b) HBV DNA standard at a high concentration (10^9 IU/mL) in the absence of HBV RNA; (c) HBV RNA standard (concentration: 5×10^8 copies/mL) in the absence of HBV DNA. As illustrated in **Figure S 4**, neither the signals of the TE buffer (a) nor the signals of the HBV DNA standard in the absence of HBV RNA (b) were detected, whereas signals of HBV RNA standard in the absence of HBV DNA (c) were detected successfully.

Next, we evaluated the condition (d) in which HBV RNA and DNA were in samples treated with or without DNase I. Results are shown in **Table S 6**. Under the conditions of HBV DNA levels from 5.7 to 8.7 $\log_{10}$ IU/mL, we compared samples treated with or

without DNase I (n=10), and no significant difference of HBV RNA was found in samples treated with or without DNase ($p>0.05$, **Table S 6**). In conclusion, four experiments above suggest that the RNA detection approach could successfully detect signals of HBV RNA no matter whether samples are treated with DNase I or not, and HBV RNA detection is unlikely influenced by the high concentration of HBV DNA.

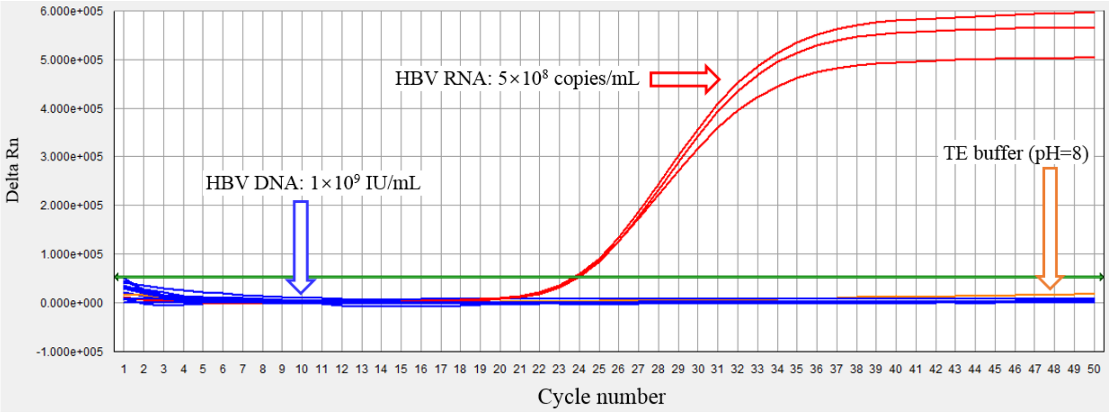

Figure S 4. Amplification curves for the validation of HBV RNA detection. (a) An orange line shows the results for TE buffer (pH=8.0) in the absence of HBV DNA or RNA (one sample was tested). (b) Twenty blue lines show results of HBV DNA samples (concentration: 10^9 IU/mL) in the absence of HBV RNA (20 samples were tested). (c) Three red lines show the results of HBV RNA standard (concentration: 5×10^8 copies/mL) in the absence of HBV DNA (3 samples were tested).

Table S 6. Detection of HBV RNA in samples harboring the high concentrations of HBV RNA and DNA treated with or without DNase I.

**Absence of DNase I**		**Treated with DNase I**		p-value (a) vs. (b)
DNA	RNA (a)	DNA	RNA (b)	
5.7	4.71±0.09*	<LOD	4.72±0.08	0.9011
6.7	5.98±0.08	<LOD	6.05±0.04	0.1252
7.7	6.93±0.01	<LOD	7.00±0.03	0.6902
8.7	8.23±0.01	<LOD	8.20±0.09	0.2616

*: Mean ± standard deviation (n=10); LOD: Limit of detection for HBV DNA is 20

IU/mL. HBV DNA and HBV RNA were quantified by log_{10} IU/mL and log_{10} copies/mL, respectively.

Limit of detection (LOD)

In order to identify the limit of detection of HBV RNA assay, we prepared three batches to test HBV RNA levels based on 7 different concentrations ranged from 100 copies/mL to 1000 copies/mL. Each batch with 40 samples was tested independently to measure the number of successful detections at different concentrations. New results are summarized in **Table S 7**. Given any serum sample, we did conduct another test if weak signals of HBV RNA could not be observed.

We performed statistical analyses to show the robustness of the HBV RNA method (**Table S 7**). Based on experimental results, HBV RNA ≥ 250 copies/mL maintained 100% (120/120) of detection rates over three batches (**Figure S 5**). With 100% of the detection rate, 250 copies/mL of HBV RNA were potentially considered as the limit of detection, though the focus of our study was not to prove the best detection limit for the HBV RNA assay.

Table S 7. Detection of HBV RNA concentrations from 100 to 1000 copies/mL.

HBV RNA	Batch 1	Batch 2	Batch 3	Mean values
1000 copies/mL	100% (40/40)*	100% (40/40)	100% (40/40)	100% (120/120)
500 copies/mL	100% (40/40)	100% (40/40)	100% (40/40)	100%(120/120)
250 copies/mL	100% (40/40)	100% (40/40)	100% (40/40)	100%(120/120)
200 copies/mL	97.5% (39/40)	100% (40/40)	100% (40/40)	99.17%(119/120)
150 copies/ml	97.5% (39/40)	100% (40/40)	97.5% (39/40)	98.33%(118/120)
100 copies/ml	97.5% (39/40)	95% (38/40)	100% (40/40)	97.5%(117/120)

*: For each concentration, 40 samples in a batch were evaluated to seek detection rates. For instance, “40/40” indicated that 40 detections were successful observed given 40 samples harboring HBV RNA. Three batches were independently tested. Note that

HBV RNA was successfully detected in all samples if HBV RNA ≥ 250 copies/mL.

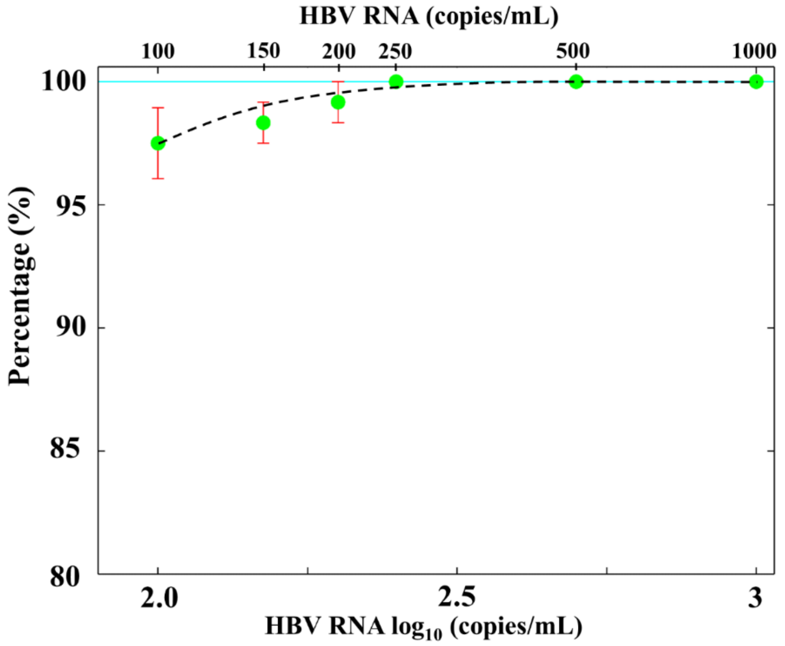

Figure S 5. Detection percentage of HBV RNA levels at the concentrations from 100 to 1000 copies/mL. For each concentration, 40 samples in a batch were evaluated to seek successful detection (also see **Table S 7**).

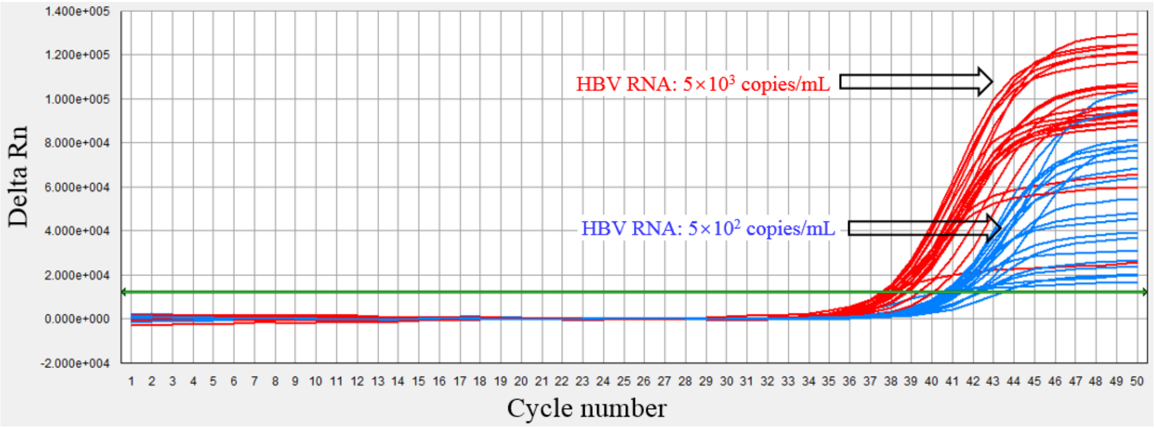

Figure S 6. Evaluation of the lower limit of HBV RNA quantification assay. HBV RNA assays were evaluated using HBV RNA specimens at the concentration of 5×10^2 and 5×10^3, whose results were illustrated in red and blue, respectively. Tests upon each specimen were repeated for 20 times. Coefficients of variability for HBV RNA at concentrations of 5×10^2 and 5×10^3 were 2.09% and 1.56%, respectively.

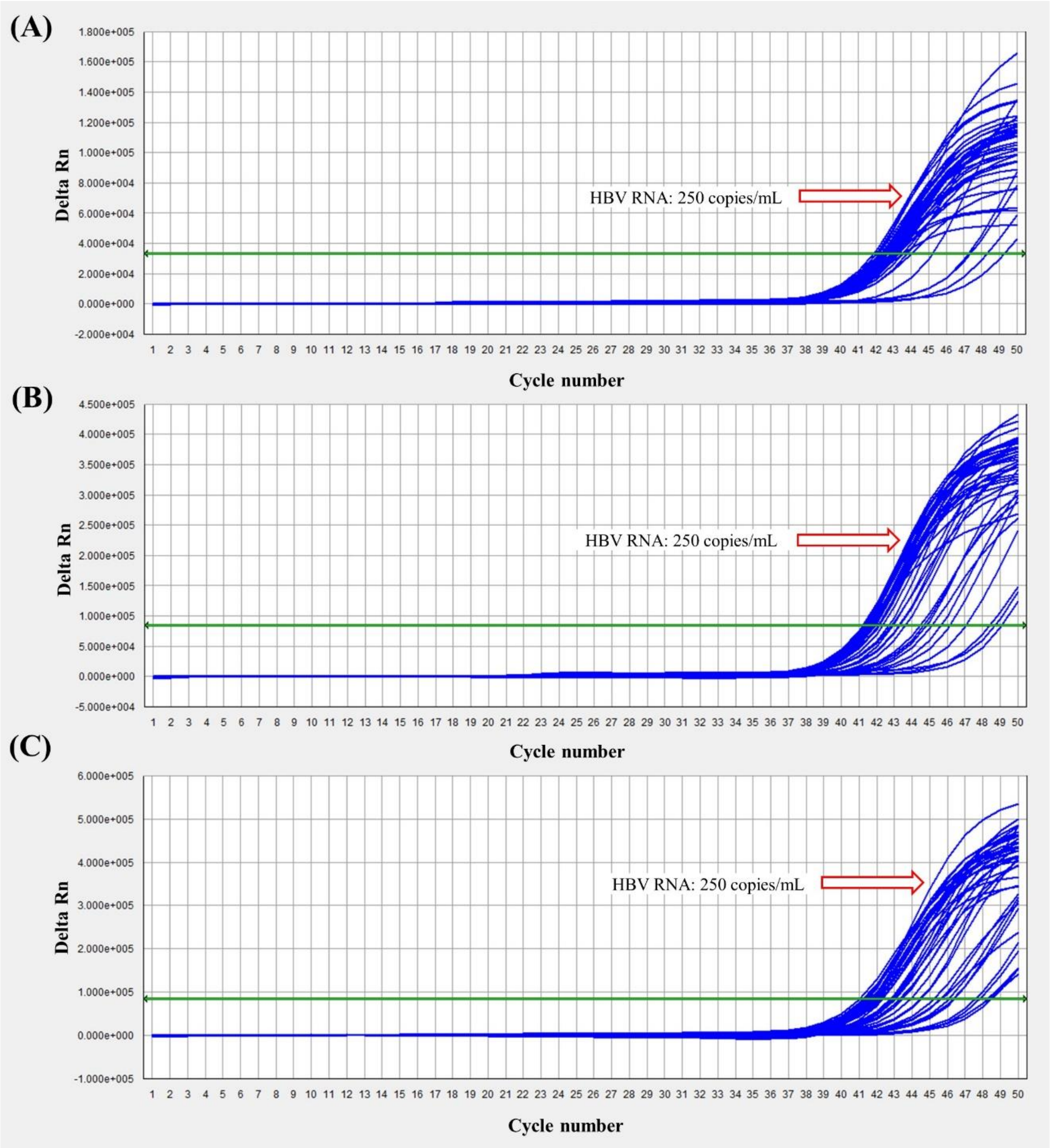

Figure S 7. Amplification curves of HBV RNA samples at the concentration of 250 copies/mL. Three batches of 40 HBV RNA samples were tested independently. Based on the calculation of the coefficient of variability (CV), the CV value of specimens harboring HBV RNA at the concentration of 250 copies/mL was 4.84%. Raw Ct data of HBV RNA with 250 copies/mL are provided at the section of Supplementary Data.

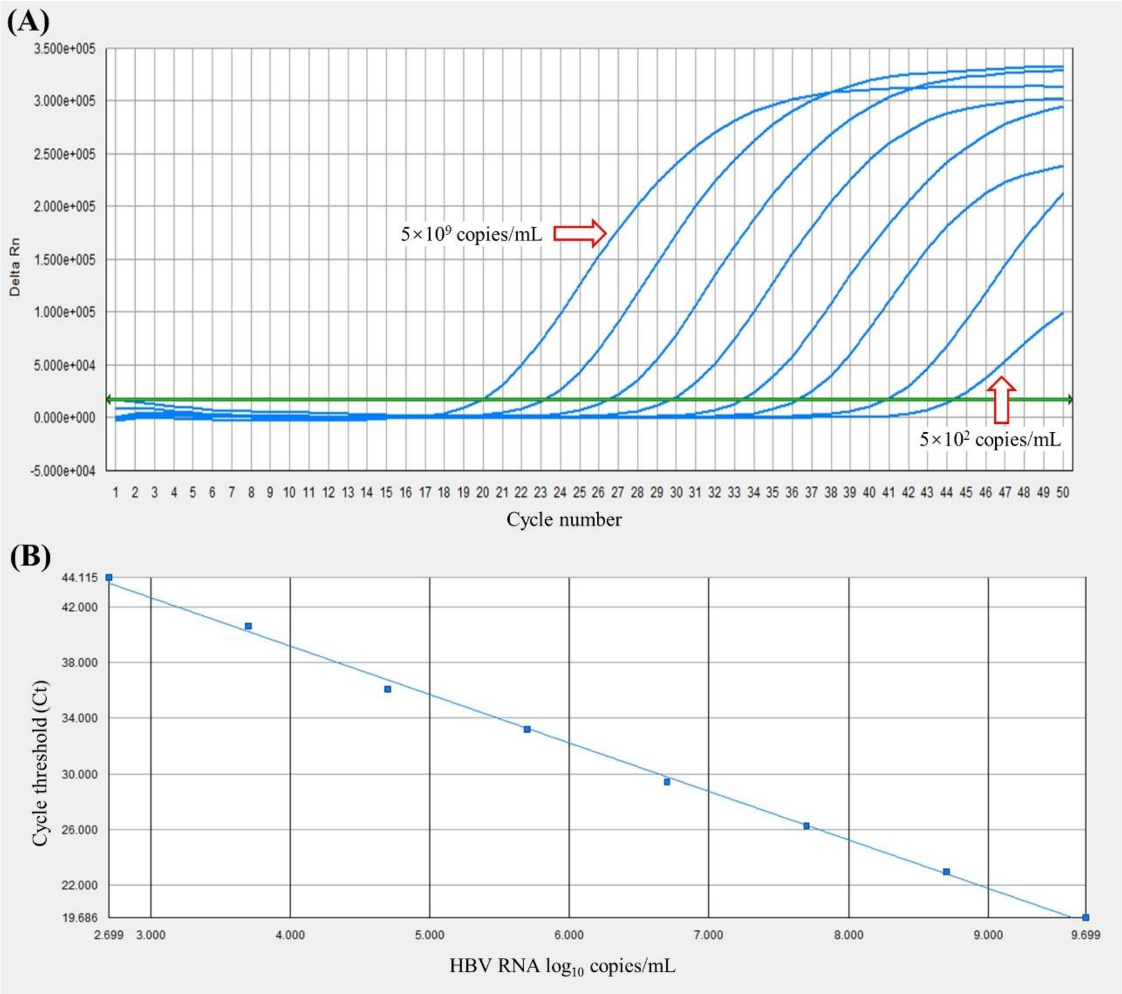

Figure S 8. Amplification curves and correlation coefficients in the HBV RNA quantification assay. (A) Amplification curves of HBV RNA that were measured by the replication cycles versus delta Rn. Amplification curves show the results of HBV RNA at the concentrations from 5×10^2 to 5×10^9 copies/mL. **(B)** Correlations between the cycle threshold (Ct) and the concentration of HBV RNA. The x-axis indicates the $\log_{10}$ values of HBV RNA concentrations from 5×10^2 copies/mL to 5×10^9 copies/mL, while the y-axis shows the cycle threshold. The linear regression curve maps the standard curve along with HBV RNA in the concentrations from 5×10^2 copies/mL ($\log_{10}$ value: 2.699) to 5×10^9 copies/mL ($\log_{10}$ value: 9.699). The correlation coefficient was -3.479002 with R^2=0.999. Note that delta Rn value is the Rn value of experimental reaction minus the Rn value of the baseline signal. Cycle threshold (Ct) is the intersection between an amplification curve and a threshold line.

For HBV RNA at 250 copies/mL, we also demonstrated the amplification curves and characterized the coefficient of variation, defined by the ratio of the standard deviation to the mean. Given 120 individual tests, the CV value of specimens harboring HBV RNA at concentrations of 250 copies/mL was 4.84% (see **Figure S 7**, PCR raw data is available in the supplementary file). Moreover, the CV values of HBV RNA at 5×10^2 and 5×10^3 were 2.09% and 1.56%, respectively (**Figure S 6**).

In addition to the analysis of HBV RNA at 250 copies/mL, we demonstrated the amplification curves of HBV RNA at the concentrations from 5×10^2 to 5×10^9 copies/mL (**Figure S 8**). The linear regression curve mapped the standard curve along with the HBV RNA concentrated from 5×10^2 copies/mL ($\log_{10}$ value: 2.699) to 5×10^9 copies/mL ($\log_{10}$ value: 9.699). The correlation coefficient was -3.479002 with R^2=0.999. Taken together, we observed a good linear relation in standard curves of HBV RNA based on diluted plasmids from 5×10^2 to 5×10^9 copies/mL.

References

1. Hou FQ, Yin YL, Zeng LY, Shang J, Gong GZ, Pan C, Zhang MX, Yin CB, Xie Q, Peng YZ, et al: **Clinical effect and safety of pegylated interferon-alpha-2b injection (Y shape, 40 kD) in treatment of HBeAg-positive chronic hepatitis B patients.** *Chinese Journal of Hepatology* 2017, **25:**589-596.
2. Sun J, Ma H, Xie Q, Xie Y, Sun Y, Wang H, Shi G, Wan M, Niu J, Ning Q, et al: **Response-guided peginterferon therapy in patients with HBeAg-positive chronic hepatitis B: A randomized controlled study.** *J Hepatol* 2016, **65:**674-682.
3. Liaw YF, Jia JD, Chan HL, Han KH, Tanwandee T, Chuang WL, Tan DM, Chen XY, Gane E, Piratvisuth T, et al: **Shorter durations and lower doses of peginterferon alfa-2a are associated with inferior hepatitis B e antigen seroconversion rates in hepatitis B virus genotypes B or C.** *Hepatology* 2011, **54:**1591-1599.
4. Lau GK, Piratvisuth T, Luo KX, Marcellin P, Thongsawat S, Cooksley G, Gane E, Fried MW, Chow WC, Paik SW, et al: **Peginterferon Alfa-2a, lamivudine, and the combination for HBeAg-positive chronic hepatitis B.** *N Engl J Med* 2005, **352:**2682-2695.
5. Cooksley WG, Piratvisuth T, Lee SD, Mahachai V, Chao YC, Tanwandee T, Chutaputti A, Chang WY, Zahm FE, Pluck N: **Peginterferon alpha-2a (40 kDa): an advance in the treatment of hepatitis B e antigen-positive chronic hepatitis B.** *J Viral Hepat* 2003, **10:**298-305.
6. Piratvisuth T, Lau G, Chao YC, Jin R, Chutaputti A, Zhang QB, Tanwandee T, Button P, Popescu M: **Sustained response to peginterferon alfa-2a (40 kD) with or without lamivudine in Asian patients with HBeAg-positive and HBeAg-negative chronic hepatitis B.** *Hepatol Int* 2008, **2:**102-110.
7. Wang YC, Yang SS, Su CW, Wang YJ, Lee KC, Huo TI, Lin HC, Huang YH: **Predictors of response to pegylated interferon in chronic hepatitis B: a real-world hospital-based analysis.** *Sci Rep* 2016, **6:**29605.
8. Janssen HL, van Zonneveld M, Senturk H, Zeuzem S, Akarca US, Cakaloglu Y, Simon C, So TM, Gerken G, de Man RA, et al: **Pegylated interferon alfa-2b alone or in combination with lamivudine for HBeAg-positive chronic hepatitis B: a randomised trial.** *Lancet* 2005, **365:**123-129.
9. Buster EH, Flink HJ, Cakaloglu Y, Simon K, Trojan J, Tabak F, So TM, Feinman SV, Mach T, Akarca US, et al: **Sustained HBeAg and HBsAg loss after long-term follow-up of HBeAg-positive patients treated with peginterferon alpha-2b.** *Gastroenterology* 2008, **135:**459-467.
10. Gish RG, Given BD, Lai CL, Locarnini SA, Lau JY, Lewis DL, Schluep T: **Chronic hepatitis B: Virology, natural history, current management and a glimpse at future opportunities.** *Antiviral Res* 2015, **121:**47-58.
11. Lok AS, McMahon BJ: **Chronic hepatitis B: update 2009.** *Hepatology* 2009, **50:**661-662.
12. van Bommel F, Bartens A, Mysickova A, Hofmann J, Kruger DH, Berg T, Edelmann A: **Serum hepatitis B virus RNA levels as an early predictor of hepatitis B envelope antigen seroconversion during treatment with polymerase inhibitors.** *Hepatology* 2015, **61:**66-76.
13. Cheng Y, Niu J, Zhang Y, Huang J, Li Q: **Preparation of His-tagged armored RNA phage particles as a control for real-time reverse transcription-PCR detection of severe acute respiratory syndrome coronavirus.** *J Clin Microbiol* 2006, **44:**3557-3561.

14. Huang X, Deng Z, Long L, Chen J, Tan D, Zhu L, Fan X, Shen T, Lu F: **Traceability, reproducibility and clinical evaluation of Sansure Realtime HCV RNA assay.** *BMC Infect Dis* 2016, **16:**47.
15. Pasloske BL, Walkerpeach CR, Obermoeller RD, Winkler M, DuBois DB: **Armored RNA technology for production of ribonuclease-resistant viral RNA controls and standards.** *J Clin Microbiol* 1998, **36:**3590-3594.
16. WalkerPeach CR, Winkler M, DuBois DB, Pasloske BL: **Ribonuclease-resistant RNA controls (Armored RNA) for reverse transcription-PCR, branched DNA, and genotyping assays for hepatitis C virus.** *Clin Chem* 1999, **45:**2079-2085.
17. Song L, Sun S, Li B, Pan Y, Li W, Zhang K, Li J: **External quality assessment for enterovirus 71 and coxsackievirus A16 detection by reverse transcription-PCR using armored RNA as a virus surrogate.** *J Clin Microbiol* 2011, **49:**3591-3595.
18. Wang J, Shen T, Huang X, Kumar GR, Chen X, Zeng Z, Zhang R, Chen R, Li T, Zhang T, et al: **Serum hepatitis B virus RNA is encapsidated pregenome RNA that may be associated with persistence of viral infection and rebound.** *J Hepatol* 2016, **65:**700-710.
